# The impact of empowering community physicians to prescribe vaccines on public vaccination: a grounded theory interview study

**DOI:** 10.3389/fpubh.2026.1734121

**Published:** 2026-02-16

**Authors:** Liqun Yao, Hailian Wang, Hong Guo, Yanping Song, Fang Yuan, Yuanyuan Wang, Jun Wei, Peng Peng, Wei Chen, Yingxin Wang, Huiyue Zhou

**Affiliations:** 1 Weifang Municipal Center for Disease Control and Prevention, Weifang, Shandong, China; 2 Fangzi District Center for Disease Control and Prevention, Weifang, Shandong, China; 3 Anqiu City Center for Disease Control and Prevention, Weifang, Shandong, China; 4 Qingzhou City Center for Disease Control and Prevention, Weifang, Shandong, China; 5 Ninth People’s Hospital Zhengzhou, Zhengzhou, Henan, China

**Keywords:** grounded theory, policy, public health, qualitative, vaccine

## Abstract

**Objective:**

Vaccination plays a crucial role in reducing the incidence and mortality of infectious diseases. However, the lack of convenient vaccination service conditions hinders vaccination rates. Weifang City in China has implemented a Vaccine Health Education Prescription (VHEP) policy, enabling qualified community physicians to provide immunization health services, including the issuance of vaccination prescriptions, to patients. This study explores the current implementation status and impact of the VHEP, aiming to further refine the policy and enhance the preventive vaccination health service model.

**Methods:**

Conducted one-on-one semi-structured interviews with 11 managers and 20 community physicians. Based on grounded theory, a theoretical framework of influencing factors for VHEP was constructed.

**Results:**

The VHEP policy encountered several challenges, including limited resources for implementers, low enthusiasm for participation among residents, and insufficient training mechanisms. Its effectiveness was contingent upon four fundamental elements: participant state, management mechanism, capacity building, and development foundation. The primary aspects of enhancing the service quality of the VHEP policy encompassed cognition, input, and guarantee.

**Conclusion:**

Authorizing community physicians to prescribe vaccines can significantly improve vaccination rates. Furthermore, enhancing individual awareness, increasing resource investment, and refining relevant policies can further promote the effective implementation of the VHEP.

## 1 Introduction

Although vaccination is recognized as one of the most effective interventions against infectious diseases in reducing the risk of illness (1, 2), global vaccination coverage has remained suboptimal over the past decade (3). Vaccine hesitancy, characterized by delay in acceptance or refusal of vaccines, poses a direct challenge to improving vaccination coverage (4).

Over the past few decades, the Chinese government has achieved remarkable success in childhood immunization through compulsory immunization policies and systematic investments. For instance, enrollment in kindergartens and primary schools requires the presentation of a complete vaccination certificate (5). Furthermore, a nationwide network of vaccination units has been established alongside a comprehensive immunization information system, which facilitates service delivery and ensures accurate vaccination records (6). However, socioeconomic and health system development has been uneven across regions, resulting in significant disparities in vaccination service access and capacity. For instance, many grassroots vaccination units face challenges such as insufficient infrastructure, staff shortages, and low educational attainment among vaccinators (7). These systemic issues are compounded by low public awareness of vaccine importance, concerns about side effects, and various sociocultural barriers (8).

The fundamental and systemic barrier resides in the operational siloing of vaccination services from general healthcare delivery within the health system. This institutional fragmentation not only disconnects immunization from longitudinal patient care but also creates a persistent service delivery gap. This gap systematically constrains clinical opportunities for healthcare providers to identify eligible individuals, initiate informed recommendations, or conveniently administer vaccines during routine consultations. This disconnect contributes to the persistently low vaccination rates among adults in China for diseases such as influenza and pneumonia (9). Addressing this coverage gap is particularly urgent in the context of an aging population and a growing burden of chronic diseases (10). To address this, Shandong Province launched a pioneering adult “Vaccine Prescription” program in August 2023, selecting five cities including Weifang for the initial pilot (11). Building on their extensive experience in immunization services, infrastructure, and policy implementation, these cities were well-positioned to lead this initiative. Unlike the traditional immunization institution-centered model, the VHEP initiative addresses health system-level constraints by implementing a series of innovative measures aimed at facilitating immunization services. These measures include: (a) expanding the scope of authorized vaccine prescribers to encompass general practitioners and relevant specialists; (b) integrating risk assessment, education, prescription, and vaccination into a cohesive closed-loop service; and (c) enhancing accessibility through both fixed-site clinics and mobile delivery options. Additionally, mobile vaccination services and home visits are also permissible under this model. Taking Weifang City as an example, the region has implemented the VHEP policy. This policy involves training general practitioners and relevant specialists, authorizing them to recommend targeted vaccination services for patients (12). Through this approach, healthcare providers can offer vaccination advice and health education while delivering medical services, taking into account the patient’s health status and actual needs (13). After receiving a vaccine health education prescription from the doctor, patients can directly proceed to the vaccination room with the prescription to complete their immunization.

By empowering frontline clinicians to assess risks, provide education, and prescribe vaccines, the VHEP has facilitated a significant transition from a passive, clinic-centered model to an active, clinician-driven approach to adult vaccination. However, the success of this innovative policy is contingent upon its effective implementation by frontline healthcare workers. Current research on adult vaccination in China predominantly addresses public vaccine hesitancy or macro-level policy analysis, leading to a considerable knowledge gap regarding the perspectives of those responsible for implementing these policies. At present, there is a lack of systematic understanding concerning how policy implementers perceive, adopt, and exercise their new prescription authority, as well as the barriers they face throughout this process.

To address this gap, this study employed a grounded theory approach to investigate the early implementation of the VHEP policy. This approach was suitable for investigating understudied, process-oriented phenomena in real-world settings. It facilitated the direct development of theories based on the experiences of policy implementers, thereby capturing the intricate mechanisms that influence early adoption. The research aimed to explore two key questions: (1) What challenges existed in the implementation of VHEP? (2) What underlying mechanisms influenced the implementation of the VHEP policy? By constructing a theory based on the experiences of implementers, this study seek to provide crucial evidence for optimizing this innovative model and supporting its potential for scaling up.

## 2 Methods

### 2.1 Setting and participants

This study was conducted from January to April 2024 in Weifang High-tech Zone and Changle, representing urban and rural areas, respectively. A purposive sampling method was employed to select administrators and physicians from primary healthcare institutions participating in the VHEP (14). Administrators comprised hospital directors, department heads, and key project leaders directly involved in VHEP-related management and decision-making. Physicians included general practitioners, specialists, and public health physicians, all of whom had received formal VHEP training and had been continuously engaged in the program since its implementation, ensuring thorough familiarity with its operations. The selection of participants followed a three-stage process: First, an initial list of eligible candidates was compiled based on recommendations from health administration departments and institutional leaders. Second, stratified sampling was applied according to institution type (urban vs. rural), professional position, years of service, and specific responsibilities to ensure diversity in roles and experiences. Finally, potential interviewees were contacted via telephone or in person to explain the study aims and procedures, after which their participation was confirmed. All participants were informed about the study and provided their voluntary consent to participate. The selection process for information providers, along with data collection and analysis, was iterative. The absence of new categories emerging in three consecutive interviews was regarded as an indication of theoretical saturation (15). Ultimately, we completed a total of 31 interviews.

### 2.2 Data collection

The interview checklist for this study was developed using a semi-structured interview guide, informed by relevant literature (16, 17). It encompassed participants’ experiences, perspectives on policy implementation, current challenges, and expectation, details were shown in Table 1. Prior to the interviews, participants were informed about the purpose and significance of the study. The location for each interview was selected based on the participants’ preferences, with the primary requirement being a quiet and private environment. All interviews were conducted in a one-on-one, face-to-face format, and the entire process was audio-recorded. Each interview lasted between 30 and 40 min. The study adhered to the principles outlined in the Declaration of Helsinki and complied with the Standards for Reporting Qualitative Research (COREQ-32) (18).

**Table 1:** Interview list.

Interview list
1. What are your primary responsibilities in relation to vaccine prescription within this program?
2. How are vaccine prescription policies formulated, tasks allocated, and implementation processes organized in your context?
3. What challenges have you encountered while carrying out vaccine prescription duties? What do you perceive as the underlying causes of these challenges?
4. Based on your involvement, what key experiences or insights have you gained from participating in vaccine prescription initiatives?
5. What achievements were realized during the vaccine prescription pilot phase, and to what extent were initial expectations met? What shortcomings were observed, and what factors do you believe contributed to them?
6. What innovative or exemplary measures has your unit implemented to promote vaccine prescription? How would you evaluate the implementation quality and effectiveness of these measures?
7. What suggestions do you have for further advancing vaccine prescription practices in the future?
8. Following the implementation of the vaccine prescription policy, what significant impacts do you anticipate it will have on the future development of vaccination in your district/county (development zone)?

### 2.3 Data analyses

**Figure 1:**
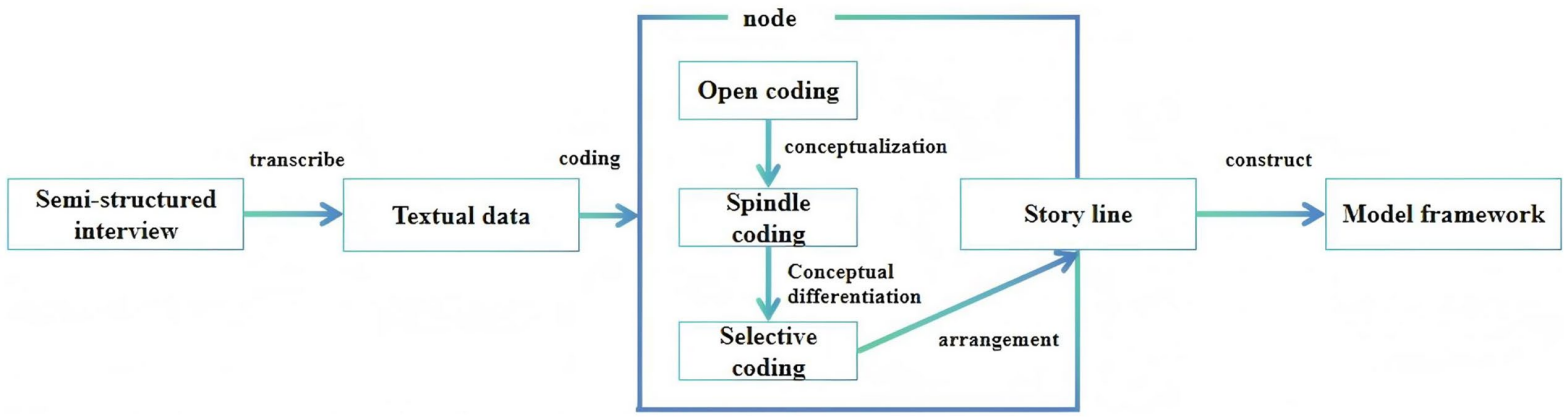
Data collection and analysis process based on grounded theory.

Interview data were analyzed in accordance with the coding procedures outlined by Corbin and Strauss, which consisted of three stages: (1) open coding, (2) axial coding, and (3) selective coding (19). Figure 1 presents the data collection and analysis process.

Open coding is also called initial coding. It entails disaggregating the raw interview transcripts, followed by the condensation and categorization of meaningful segments through iterative comparison, ultimately leading to the conceptualization of initial themes (20). In this study, two researchers independently reviewed all interview transcripts, highlighting statements related to VHEP and eliminating sentences that conveyed similar, identical, or ambiguous meanings. The remaining sentences were then preliminarily categorized to establish the original concepts and classification results for open coding.

Axial coding involved systematically refining, adjusting, and classifying the categories derived from open coding. This process included merging categories with shared meanings and clarifying the relationships among subcategories to establish coherent connections (21). Given the influence of human, material, and environmental factors on the implementation of health policies, the researchers integrated the initial categories, analyzed their internal connections, and extracted the main categories based on these considerations.

Selective coding was conducted to identify and articulate the core category emerging from the data. This involved systematically analyzing its relationships with other main categories, integrating these connections into a coherent narrative (“storyline”), and ultimately deriving the theoretical findings of the study (22). Guided by the theme of the VHEP, this study synthesized raw textual statements and the main categories derived from axial coding to identify a core category. Through this process, this study delineated the current implementation status, underlying causes, and potential strategies related to the policy, thereby constructing a coherent theoretical “storyline.”

### 2.4 Theoretical saturation

Theoretical saturation refers to the point at which no additional data can be obtained to further develop the properties of a category, serving as a critical indicator of the completeness of the theoretical model (23). In this study, theoretical saturation was assessed through a combination of expert interviews and validation processes. The procedure involved two steps: (a) Three experts in the relevant field were interviewed, and their responses were transcribed and analyzed through coding. The analysis indicated that no new codes emerged beyond those identified in previous coding rounds. (b) The research data, coding results, and their alignment with the CFIR framework were subsequently submitted to experts for review. Consensus among the experts confirmed that the theoretical model had achieved data saturation.

### 2.5 Reflexivity and positionality

To meet the rigor requirements of qualitative research, this study controlled for potential influences stemming from the backgrounds and perspectives of the research team on the study. The team consisted of three experts: two senior public health researchers specializing in vaccine policy and one master’s-level researcher focused on health services research and implementation science. All members possessed experience with qualitative methods and were well-acquainted with China’s primary healthcare context. This background facilitated a nuanced understanding of policy implementation and system-level barriers; however, it also introduced potential biases, particularly concerning structural and administrative challenges. To mitigate such biases, we maintained reflective logs throughout data collection and analysis to document and delineate our preconceptions. Furthermore, data coding was conducted independently by two researchers, with discrepancies discussed and resolved through consensus and regular consultations with an external qualitative methods expert. To enhance the credibility of these findings, the summary of the results was shared with selected participants for verification. Following the completion of the grounded theory analysis, the Consolidated Framework for Implementation Research (CFIR) was used as a post-hoc sensitizing and interpretive lens (24). The Consolidated Framework for Implementation Research (CFIR) is used to assess the diverse factors influencing the implementation of complex health interventions. Previous studies have demonstrated its strong applicability in policy implementation research, as well as its capacity to systematically categorize barriers and facilitators (25).

### 2.6 Ethics statement

This research obtained ethical approval from the Ethics Committee of the Weifang Center for Disease Control and Prevention (ID: 2024-003). Respondents were provided with information regarding this study and gave their written or oral consent prior to the interview. All participants were assured of the following: (a) their right to withdraw from the study at any time; (b) that participant information would be limited to use within this study.

## 3 Results

### 3.1 Sociodemographic characteristics

A total of 31 interviewees participated in the study, comprising 11 administrators (4 females and 7 males) and 20 physicians (8 females and 12 males). The average age of all interviewees was 49.52 years, and all had over 10 years of work experience. 12 individuals had an academic background in Internal Medicine, 6 in Public Health, 6 in Preventive Medicine, 4 in Public Administration, and 3 in Nursing (shown in Table 2).

**Table 2:** Demographic characteristics of participants.

Category	Subcategory	*N* = 32
Interviewee’s role		
	Manager	11
	Physician	20
Gender		
	Female	12
	Male	19
Age (years)		
	30–39	3
	40–49	7
	50–59	21
Educational level		
	Junior college	2
	Secondary specialized school	1
	University or above	28
Position		
	Intermediate Professional Title	17
	Associate Senior Professional Title	12
	Senior Professional Title	2
Academic background		
	Preventive medicine	6
	Public Health	6
	Internal Medicine	12
	Nursing	3
	Public administration	4
Project responsibilities		
	Evaluation and feedback	6
	Monitoring and supervision	7
	Prescription and health promotion	13
	Program development	5
	Project responsibilities	1
Years of work		
	≤10	5
	11–19	5
	20–29	22

### 3.2 Three-stage coding

A total of 32 original concepts related to the VHEP were extracted from the original statements, which were subsequently consolidated into eight open codes, i.e., limitations in implementer resources, low enthusiasm among residents, inadequate training mechanisms, poor cooperation mechanisms, lagging service content, insufficient effectiveness, information silos, policies to be strengthened. Based on open coding and the connotation mining of original concepts, four axial codes were identified: participants, management mechanisms, capacity building, and development foundation. Through the study and synthesis of the relationships between open codes and axial codes, three selective coding were established: cognition, input, and guarantee. The specific coding analysis process was illustrated in Table 3.

**Table 3:** Coding analysis.

Selective coding (*N* = 3)	Axial coding (*N* = 4)	Open coding (*N* = 8)	Original concepts (*N* = 32)
Cognition	Participant state	1. Limitations in implementer resources	The shortage of personnel
			Low professional competence
			Low sense of identity
			Limited service capacity among personnel
			Cumbersome software system
			Occupational Prejudice
		2. Low enthusiasm among residents	Low trust in vaccines
			Backward concept
			Passive demand
			High vaccination cost
			Pseudoscientific information
			Policy authority
Input	Management mechanism	3. Inadequate training mechanisms	Solidification of the training form
			Poor training effect
			Insufficient training time
			Low constraints
			Lack of feedback channels
		4. Poor cooperation mechanisms	Cross-Regional Collaboration Barriers
			Insufficient Integration of Medical and Preventive Services
			Lack of Multi-Department Coordination
			Absence of Substantive Support
	Capacity building	5. Lagging service content	Insufficient supply of vaccines
			Single service offering
		6. Insufficient effectiveness	Short implementation time
			Insufficient government promotion
			Unfocused promotion
Guarantee	Development foundation	7. Information silos	Independence of Information Systems
		8. Policies to be strengthened	Insufficient normative clarity in policies
			Inadequate legal safeguards
			Lack of assessment targets
			Inadequate social support
			Insufficient financial support

### 3.3 A multidimensional analysis of cognition, input, and guarantee

Cognition: The implementation process faced significant challenges due to gaps in awareness, competency, and commitment across multiple levels. Among healthcare providers, limited authorization, low self-efficacy, and role ambiguity contributed to diminished engagement. For the public, vaccine hesitancy was exacerbated by concerns regarding cost, misinformation, and a low perceived necessity for vaccination.

Input: Operational weaknesses were evident in training, collaboration, and resource allocation. Training mechanisms were characterized as brief, infrequent, and overly theoretical, which failed to foster practical confidence. Inter-departmental and cross-sector cooperation was obstructed by administrative silos. Additionally, material and financial resources were inadequate, leading to frequent vaccine shortages and a lack of additional compensation for prescribers, which adversely affected sustainability.

Guarantee: The absence of a robust policy framework, infrastructural support, and evaluative foundations posed a threat to long-term viability. Policy frameworks were perceived as exploratory and lacking essential legal safeguards, clear standards, and performance incentives, which undermined confidence among frontline workers. Information systems operated in silos, hindering integrated data tracking. The effectiveness and promotion of vaccination efforts were limited by short implementation timelines, unfocused government advocacy, and insufficient social marketing support (details as shown in Supplementary material).

**Figure 2:**
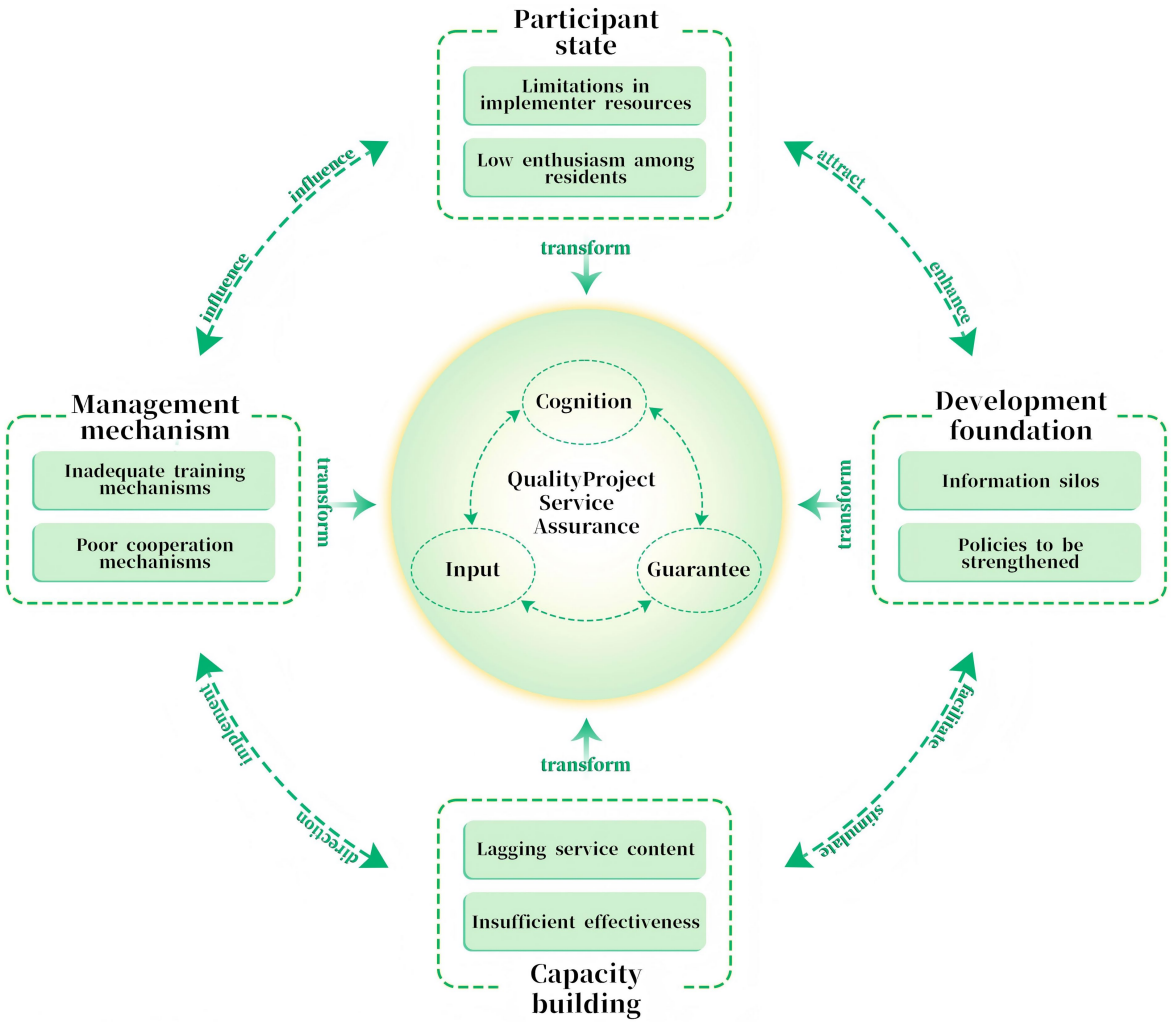
Model of the influencing factors of VHEP.

Through a three-level coding process, this study identified that the implementation of the VHEP policy was contingent upon four foundational elements: participant state, management mechanism, capacity building, development foundation. The core factors that ensured the effectiveness of the VHEP policy include cognition, input, and guarantee. Given constraints such as limitations in implementer resources, low enthusiasm among residents, inadequate training mechanisms, it was essential to enhance individual awareness, increase investment, and improve safeguards to effectively address these challenges. Through the analysis and comparison of the relationships among various codes, this study constructed a model of influencing factors for the VHEP, as shown in Figure 2.

## 4 Discussion

This study, based on semi-structured interviews with managers and community physicians, developed a model of factors influencing the VHEP, organized around three core dimensions: cognition, input, and guarantee. The findings suggested that improving VHEP implementation requires integrated efforts across four fronts: enhancing participant cognition, strengthening management mechanisms, building service capacity, and solidifying development foundations. The study further proposed a novel policy-collaboration model that links VHEP with broader medical prevention strategies. By identifying key barriers during VHEP rollout, this research offered practical insights for enhancing immunization policy effectiveness in community and rural settings.

### 4.1 The human core: cognition as the primary driver and barrier

This study identified that community physicians’ awareness and attitudes toward the VHEP policy shaped its implementation outcomes. A notable barrier frequently reported by frontline physicians was their reluctance toward the newly mandated task of “authorized prescription-writing.” This reluctance was attributed to existing workforce shortages, high routine workloads, and the perceived additional burden—concerns that were particularly acute in rural settings, where human resources were described as especially strained (26). Administrators, while acknowledging workload issues, more often emphasized the necessity for a change in attitude and professional commitment. Physicians’ engagement was further influenced by self-reported limitations in knowledge and confidence regarding vaccine indications, a theme that resonated with both urban and rural participants, but was often linked by rural physicians to fewer prior training opportunities. On the demand side, participants acknowledged that residents’ willingness to get vaccinated remains low, with practical considerations being that people perceive themselves as having low health literacy and tend to adopt a passive attitude toward acceptance (27). Urban physicians more frequently cited residents’ concerns about cost and vaccine safety, whereas rural providers highlighted deeper accessibility barriers and stronger influences of local community beliefs. These reported differences underscore the necessity for tailored strategies to enhance “cognition”: empowering physicians may require differentiated support for rural versus urban practitioners, while public engagement strategies need to address locally salient concerns (28).

### 4.2 The operational layer: input factors that translate policy into practice

This study revealed that resource investment—particularly through the two dimensions of management mechanisms and capacity building—significantly shaped the quality and effectiveness of prescription policy implementation. Regarding management mechanisms, VHEP training was widely described by participants as suffering from uniform formats, ambiguous content, a lack of continuity, and insufficient frequency. These identified shortcomings collectively pointed to the need for establishing a sustainable training system with clear objectives, specific content, and a blended online-offline model to support ongoing professional development (29). Concurrently, the absence of a robust evaluation mechanism was notable: because VHEP was not included in the performance standards of primary health institutions, assessment indicators remained vague, and effective incentives were lacking. This not only hindered physicians’ ability to reasonably gauge their workload but also dampened their motivation for clinical promotion. Therefore, it was imperative to develop a standardized and transparent evaluation framework that linked assessment outcomes with appropriate incentives, thereby enhancing engagement at the grassroots level (30). Furthermore, cross-regional collaboration was hampered by inefficient information-exchange channels, leading to unclear responsibilities and implementation conflicts. Establishing a unified project-management team was identified as necessary to clarify roles and improve coordination among stakeholders (31). At the level of capacity building, the program was found to be overly focused on prescription issuance, while extended services such as vaccine science communication and health management remained underdeveloped. Coupled with an unstable supply of non-program vaccines, these limitations constrained service accessibility and the program’s ability to comprehensively address residents’ health needs (32). Program outreach relied predominantly on traditional offline methods, which proved insufficient to effectively cover rural areas. Accordingly, integrating VHEP into long-term health benefit policies and actively utilizing diversified channels—including social media—for science communication were recommended to extend the program’s reach and sustainability (33).

### 4.3 The foundational layer: development guarantees for long-term viability

This study found that a favorable social and cultural environment, along with policy support, served as the foundational basis for the effectiveness of the VHEP, which aligned with previous research (34). All participants reported that the VHEP enhanced residents’ awareness of disease prevention and increased their motivation to receive vaccinations. However, individual vaccination failures might lead individuals to forgo subsequent vaccinations (35). In recent years, concerns regarding vaccine safety, anti-vaccination sentiments, and the proliferation of pseudoscientific information about vaccines had exacerbated public anxiety and skepticism, resulting in polarized perceptions and judgments regarding vaccine risks, ranging from complete trust to total denial (36, 37). To address this issue, the government must adopt proactive measures to disseminate scientific information and counter misinformation, thereby restoring public confidence in preventive immunization (38). A critical finding was the perceived lack of legal and policy safeguards for prescribers. Physicians across all sites expressed concerns about potential liability, which made them cautious in promoting vaccines. Administrators acknowledged this as a significant policy gap that requires higher-level resolution. This shared concern among various groups underscores a non-negotiable prerequisite for scale-up: clear legal frameworks and liability protections are essential to secure buy-in from frontline providers. Finally, in terms of future scalability, the research findings indicate that integrating the program into the routine public health system and ensuring stable financial support are critical steps for transforming the VHEP from a project-based initiative into a sustained service (39).

## 5 Limitations

To the best of our knowledge, this study represents the first grounded theory investigation in China concerning the current status and challenges faced by authorized community physicians in vaccine prescription. Our findings offer significant insights for the further implementation of the VEHP policy and the expansion of immunization coverage. Nonetheless, this study has certain limitations. Firstly, as a qualitative investigation, the results are inevitably subject to researchers’ interpretation, despite our efforts to maintain rigor through iterative coding and expert consultation. Secondly, this study investigated the challenges of policy implementation in both urban and rural settings from the perspectives of physicians and administrators. However, it did not fully examine the variations in perspectives across different participant groups and regional contexts. Furthermore, the exclusion of residents from the study presents a notable gap. Future studies should incorporate residents as a research population and examine differences in perspectives across diverse groups in various contexts. Finally, the study was conducted during the initial pilot phase in selected urban and rural areas of one province. The relatively short implementation timeframe and contextual specificities—such as local institutional capacities, socioeconomic conditions, and existing immunization service structures—may limit the direct transferability of all findings to other regions in China or to different adult vaccination initiatives. As the policy expands, more sophisticated research designs, including mixed-methods or longitudinal studies across diverse settings, will be necessary to validate and generalize the findings.

## 6 Implications

Despite these limitations, the study provides significant policy and practical implications for promoting the sustainable development of the VHEP and similar primary care initiatives. Future efforts should concentrate on three key areas: (1) Targeting ‘Cognition’: It is imperative to establish a collaborative implementation support system. Policy implementation must progress beyond isolated improvements to develop an integrated framework that aligns workforce competencies, management mechanisms, and institutional environments. This includes enhancing the professional and communication skills of frontline providers, optimizing interdepartmental coordination, and ensuring the interoperability of information systems. (2) Targeting ‘Input’: It is crucial to construct an inclusive and resilient participation network. Diverse engagement channels should be established to involve policymakers, implementers, and community residents, treating public communication as a core intervention component. Contextual adaptation will be essential when scaling the policy; factors such as regional economic disparities, health resource distribution, local governance structures, and sociocultural attitudes toward vaccination must be carefully considered to tailor strategies effectively. (3) Targeting ‘Guarantee’: Efforts must be directed toward consolidating an institutionalized and routine governance foundation. Long-term policy effectiveness relies on integrating core components into the routine public health service system and reinforcing them with legal and fiscal safeguards. This entails transitioning the VHEP from a pilot program to a standardized, institutionalized health service, supported by stable funding and clear regulatory frameworks to ensure sustainability and equitable scalability.

## 7 Conclusion

This study demonstrated that authorizing community physicians to prescribe vaccine health education expanded public access to immunization, improved health literacy, and contributed to higher vaccination rates. Public awareness, supportive policies, and adequate resources were pivotal in promoting vaccination at the grassroots level. To sustain this progress, it was essential to strengthen positive public perceptions of immunization, increase governmental investment in material and human resources, and refine policies to overcome systemic barriers. By promoting credible messaging and reducing vaccine hesitancy, these measures could further enhance residents’ voluntary uptake and sustained participation in immunization programs.

## Data Availability

The raw data supporting the conclusions of this article will be made available by the authors without undue reservation.
