# Supplementary material for: The impact of empowering community physicians to prescribe vaccines on public vaccination: a grounded theory interview study
Source: Front Public Health. 2026 Feb 16;14:1734121. doi: 10.3389/fpubh.2026.1734121 (PMC12950803; doi:10.3389/fpubh.2026.1734121)
Supplement: Supplementary file 1 [file Table_1.docx]

| **Examples of open coding analysis.** | | |
| --- | --- | --- |
| **Open coding (N=8)** | **Original concepts (N=32)** | **Original statements (Segments)** |
| 1. Limitations in implementer resources | The shortage of personnel | Currently, access has been granted exclusively to doctors in specific departments, namely respiratory and dermatology. Consequently, physicians in other specialties lack the necessary permissions. (No.1) |
|  |  | This limited authorization is particularly concerning given the existing shortage of professionals in these fields. (No.7) |
|  | Low professional competence | Many (physicians) are often completely unaware of both the contraindications and indications associated with various vaccines. (No.2) |
|  |  | I hesitant to prescribe it (vaccine) due to my limited experience in this area. (No.3) |
|  | Low sense of identity | Some medical professionals, including certain administrative staff, exhibit skepticism regarding the efficacy of vaccines. This skepticism results in a diminished initiative and enthusiasm in their efforts to promote vaccination. (No.9) |
|  | Limited service capacity among personnel | There is a high volume of patients, and the consultation time is limited. So, there is insufficient opportunity to address vaccine-related issues and provide health education regarding vaccines. (No.10) |
|  | Cumbersome software system | To implement the VHEP, a new system was installed; however, its operation presents challenges, particularly for individuals of advanced age. (No.20) |
|  |  | The software for registering VHEP-related information is overly complex. (No.16) |
|  | Occupational Prejudice | The clinical perspective suggests that vaccination is not considered a personal responsibility, but rather the duty of immunization personnel, leading to a reluctance to accept this role. (No.17) |
| 2. Low enthusiasm among residents | Low trust in vaccines | Some individuals contend that an increase in vaccinations may be detrimental to health, arguing that the advocacy for vaccine health education initiatives at the grassroots level resembles product promotion. (No.22) |
|  | Backward concept | People consider it foolish to pay for unseen effects (vaccination to prevent diseases). (No.25) |
|  | Passive demand | Many current issues stem from the fact that some residents do not perceive vaccination as an urgent personal necessity. (No.13) |
|  |  | They tend to consider treatment only when they are unwell, rather than prioritizing prevention. (No.25) |
|  | High vaccination cost | The prices of non-immunization program vaccines are excessively high and are not covered by health insurance. (No.30) |
|  | Pseudoscientific information | There is a significant amount of discourse online regarding the ineffectiveness and potential harms of vaccines, leading to a growing distrust among the general public towards vaccination. (No.29) |
|  | Policy authority | The VHEP is currently in the exploratory stage, necessitating the gradual enhancement of relevant policies and laws. (No.17) |
|  |  | There exists a pervasive distrust towards the VHEP policy, with individuals expressing skepticism regarding its accessibility to services and the assurance of follow-up support. (No.31) |
| 3. Inadequate training mechanisms | Solidification of the training form | The current methods of online training and on-site training occupy valuable work hours, primarily focusing on theoretical instruction while lacking sufficient practical application in real-world settings. (No.13) |
|  | Poor training effect | The content presented during the training lacks depth and practical relevance. (No.5) |
|  |  | The material covered is largely common knowledge, lacking specificity and failing to adequately address pertinent issues. (No.15) |
|  |  | The results of the training do not sufficiently equip doctors to meet the needs of their patients. (No.28) |
|  | Insufficient training time | The duration of each training session is typically one to two hours, and the frequency is relatively low, occurring once or twice a month. (No.3) |
|  |  | The short cycle limits the amount of knowledge that can be acquired. (No.21) |
|  | Low constraints | Some physicians frequently cite their busy work schedules as a reason for not participating in training sessions, resulting in absenteeism at each training event. (No.17) |
|  | Lack of feedback channels | In the workplace, encountering problems without knowing whom to consult can be challenging. (No.10) |
|  |  | The exchange of experiences is limited to on-site training, resulting in a lack of feedback platforms. (No.31) |
| 4. Poor cooperation mechanisms | Cross-Regional Collaboration Barriers | The procedures for urban residents from other areas to access vaccination services in our region are complex. (No.13) |
|  |  | We aim to establish convenient channels for information exchange with other cities; however, this process necessitates the involvement of medical departments, administrative agencies, and public health authorities. (No.8) |
|  | Insufficient Integration of Medical and Preventive Services | There is a lack of collaboration between clinicians and public health physicians, leading to a limited understanding of each other's work. (No.1) |
|  |  | Due to administrative structures, clinical treatment and public health preventive care function as two independent systems. (No.19) |
|  | Lack of Multi-Department Coordination | We need the help of the financial sector, the health administration and other relevant departments. (No.9, No.16) |
|  | Absence of Substantive Support | The higher authorities should not rely solely on a single policy document; rather, they must provide tangible support, such as dispatching experts for guidance or offering material assistance. (No.28) |
| 5. Lagging service content | Insufficient supply of vaccines | Certain vaccines, including those for cervical cancer, are frequently out of stock. (No.8) |
|  |  | Annually, we experience three to five instances of vaccine shortages. (No.17) |
|  |  | Residents express their concerns to us when they discover that vaccines are unavailable. (No.29) |
|  | Single service offering | Post-vaccination health management is often lacking. (No.4) |
|  |  | For certain populations affected by infectious diseases, such as individuals living with HIV, we refrain from administering vaccinations to them. (No.3) |
| 6. Insufficient effectiveness | Short implementation time | The implementation of the vaccine health education prescription is still in its early stages, and thus far, we have not observed long-term benefits. (No.1) |
|  | Insufficient government promotion | If the government can enhance its support and policy guidance for vaccine health education prescriptions, it will facilitate a more in-depth implementation of these vaccine prescriptions. (No.2) |
|  | Unfocused promotion | This policy encompasses a broad spectrum of regions and seeks to accomplish multiple objectives; however, it lacks a clear identification of its key priorities. (No.7) |
| 7. Information silos | Independence of Information Systems | The vaccination clinic system and the hospital original information system operate independently, making it impossible to track the subsequent status of patients' vaccine health education prescriptions. (No.23) |
| 8. Policies to be strengthened | Insufficient normative clarity in policies | The standards for formulating VHEP by different institutions have not yet been unified, resulting in significant discrepancies in areas such as physician prescribing practices and vaccine administration procedures. (No.18) |
|  |  | Enhancing policy standardization represents a key challenge currently faced in this field. (No.22) |
|  | Inadequate legal safeguards | Without clear legal provisions to safeguard post-authorization prescriptions, I, as a physician, am reluctant to issue them. (No.13) |
|  |  | This policy remains exploratory and lacks a legal framework to protect those who implement it. (No.9) |
|  | Lack of assessment targets | There are no regulations specifying the exact number of prescriptions. (No.13) |
|  |  | Clear acceptance criteria for projects are lacking regarding objectives such as public vaccine literacy, policy promotion efforts, and vaccination rates. (No.25) |
|  | Inadequate social support | Enhancing the public's vaccine literacy necessitates extensive publicity and science education, which requires robust support from social media platforms. Unfortunately, such support remains limited. (No.14) |
|  | Insufficient financial support | The authorization of prescriptions represents an additional responsibility assigned to us by management, and it is not compensated. (No.22) |
|  |  | In this process, we (physicians ) are not paid extra. (No.31) |
